# Lean body mass positively associate with blood pressure in Chinese adults: the roles of ages and body fat distribution

**DOI:** 10.1186/s12889-023-17312-0

**Published:** 2023-12-07

**Authors:** Wen Yuan, Yi Zhang, Li Chen, Jieyu Liu, Manman Chen, Tongjun Guo, Xinxin Wang, Tao Ma, Qi Ma, Jianuo Jiang, Mengjie Cui, Yanhui Dong, Yi Song, Jun Ma

**Affiliations:** 1 Institute of Child and Adolescent Health, School of Public Health, National Health Commission Key Laboratory of Reproductive Health, Peking University, 38 Xueyuan Rd, Haidian District, Beijing, 100191 China; 2 School of Public Health and Management, Key Laboratory of Environmental Factors and Chronic Disease Control, Ningxia Medical University, No.1160, Shengli Street, Xingqing District, 750004 China

**Keywords:** Lean body Mass, Hypertension, Blood pressure, Fat distribution, DXA, Age Trend

## Abstract

**Background:**

The relationship between lean body mass (LBM) and blood pressure (BP) is controversial and limited. This study investigated the associations between LBM indexes and BP in adults of different ages and with varying body fat distribution.

**Methods:**

The data for the present analysis was obtained from a cross-sectional survey of 1,465 adults (50.7% males) aged 18–70 years conducted in Beijing, China. Regional LBM and fat distribution, including fat mass (FM) and android to gynoid fat ratio (AOI), were assessed using a dual-energy X-ray bone densitometer. Generalized Liner Model (GLM) was employed. Confounders, including age, sex, height, weight, smoking, and alcohol use, were evaluated through questionnaires and physical examinations.

**Results:**

Males had higher rates of hypertension (11.19% vs. 4.92%) and prehypertension (21.57% vs. 14.59%) than females. The mean systolic blood pressure (SBP) and diastolic blood pressure (DBP) were 122.04 mmHg and 76.68 mmHg. There were no significant associations between LBM and DBP (*p* > 0.05). However, arms LBM (β = 1.86, 95% CI: 0.77, 2.94) and trunk LBM (β = 0.37, 95% CI: 0.01, 0.73) were significantly associated with SBP. The association of LBM on DBP was stronger with increasing ages, and stronger in females than in males (*p* < 0.001). The association between adults’ arms LBM and SBP was stronger in the high level FM group (β = 2.74 vs. β = 1.30) and high level AOI group (β = 1.80 vs. β = 2.08).

**Conclusion:**

The influence of LBM on SBP increases with age, particularly after the age twenty years in females. For adults with high FM or high AOI, LBM in the arms, showed a stronger positive predictive association with SBP. This suggests that, in addition to controlling fat content, future efforts to improve cardiovascular health in adults should include the management of LBM (especially in the upper body).

**Supplementary Information:**

The online version contains supplementary material available at 10.1186/s12889-023-17312-0.

## Introduction

Multiple studies have shown that lean body mass (LBM) or total skeletal muscle (TSM) play a key role in energy metabolism, and enhancing LBM and muscle strength has been advocated for preventing age-related weakness and sarcopenia [1–3]. However, some studies have shown that in certain populations, such as obese children, LBM is adversely associated with cardiometabolic risk factors and increases blood pressure [4, 5]. Elevated blood pressure and hypertension are major risk factors for several cardiovascular diseases (CVD), resulting in significant health and economic burdens [6]. Therefore, it is of great significance to explore the relationship between LBM and blood pressure.

Some studies have found an adverse relationship between LBM and hypertension [7–9], while other studies have found the opposite association [10, 11]. Such inconsistencies may be limited to specific populations, such as obese children [4] and postmenopausal women [12], which may restrict the usefulness of alternative measures, such as different anthropometry [13]. Therefore, it is of great significance to utilize more precise measurement methods and explore the relationship between LBM and blood pressure in a wider population.

In addition, body fat mass or fat distribution is closely related to LBM [14, 15], making it a factor worth considering. There was one study that analyzed the independent role of TSM after controlling for fat distribution [8]. Dual-energy X-ray absorptiometry (DXA) is considered the gold standard for body composition measuring, which can obtain total and local body composition content with high stability and repeatability [16]. This study will further explore the influence of LBM indexes on blood pressure changing in Chinese adults, taking into account age and sex differences and stratifying fat components, which will provide more accurate guidance for the prevention and control of hypertension in different populations.

## Materials and methods

### Participants

A cross-sectional survey was conducted among adults aged 18 to 70 in Beijing, China, in Otc, 2020. The inclusion criteria were healthy adults and to avoid including fitness enthusiasts, athletes, pregnant women, and special groups who are undergoing diet or exercise intervention, and the signed prior informed consents. Following the exclusion criteria, adults who individuals with a history of various important organs (such as cardiovascular disease, pneumonia, hepatitis, gastritis, and nephritis, etc.), abnormal physical development such as pygmyism or gigantism, physical impairments or deformities, or acute disease symptoms in the past month and had not yet recovered were excluded [17]. In addition, the project considered the establishment of a body composition equation, so a rigorous participant recruitment process was adopted, with a balanced distribution of age and BMI of the participants. Adults aged 18 and above were divided into one age group every 5 years after the age of 20, and were divided into 11 groups: 18–21 years old, 21–25 years old, 26–30 years old, 31–35 years old, 36–40 years old, 41–45 years old, 46–50 years old, 51–55 years old, 56–60 years old, 60–65 years old, and 66 years old and above. Thin, normal-weight, overweight and obese adults were included in each age group.

**Fig. 1:**
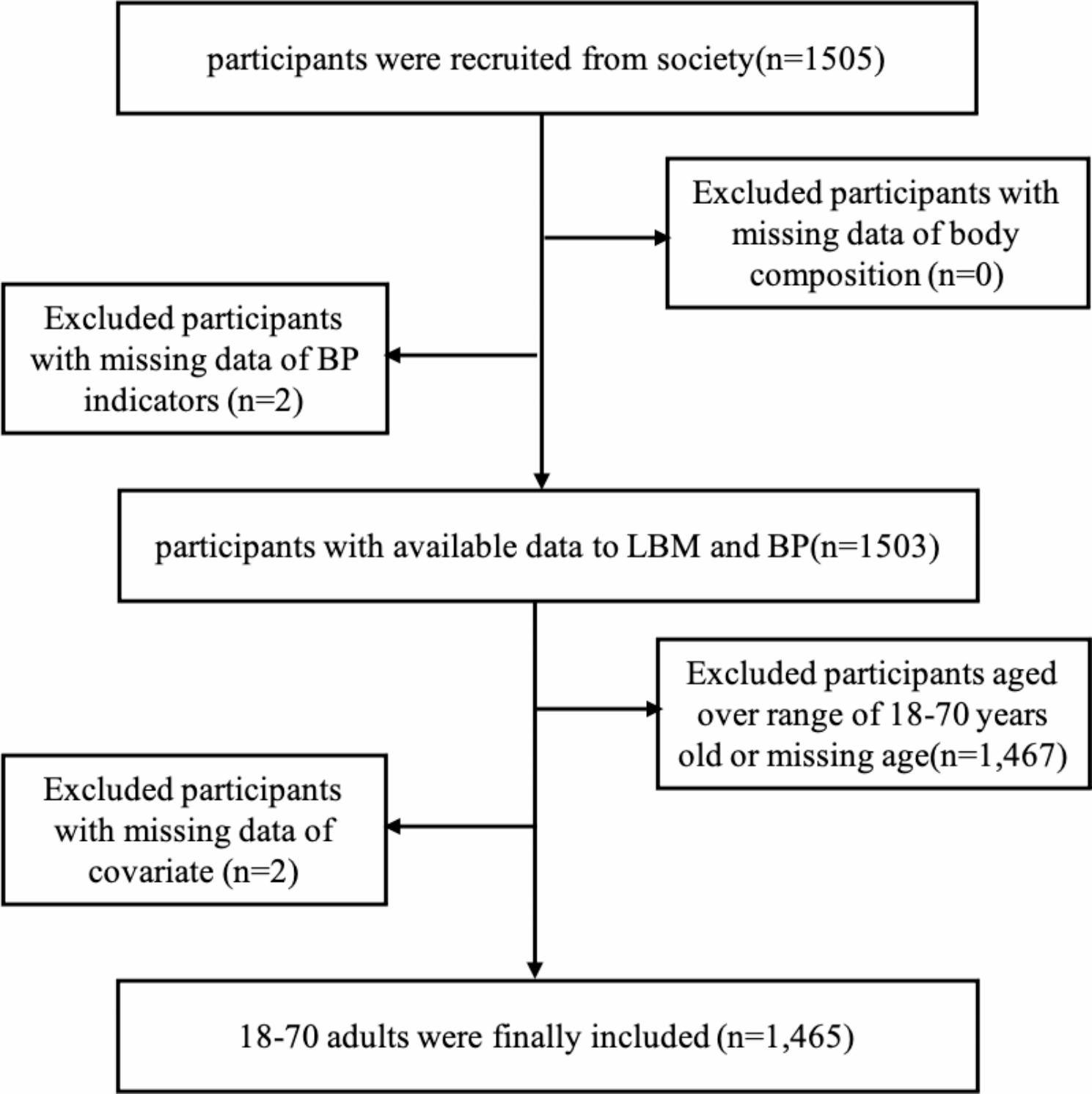
The flow chart of study population selection

As shown in Figs. 1 and 505 participants were recruited from the society. Then, individuals with missing data on body composition, blood pressure, and other covariates were further excluded. The final sample consisted of 1,465 adults, including 742 males (50.6%). The age distributions were as followed: 267 individual were 18–20 years old, 287 were 21–30 years old, 267 were 31–40 years old, 228 were 41–50 years old, 225 were 51–60 years old and 191 were 61–70 years old. The recruitment and data collection procedures were approved by the institutional review board of the affiliating university(number: IRB00001052 20,024). The research assistants provided a detailed introduction of the research purpose and content of the project to the potential adult participants. Written informed consent was obtained from all participants. The project team distributed the questionnaires to the participants before the physical examination. The research assistants collected the completed questionnaires on the day of the physical examination and handed them over to the project team members.

### Demographic variables

The participants reported their birth date, sex, and medical history. We obtained their age by subtracting their birth date from the recorded individual examine date. Height was measured using a uniform and calibrated mechanical stadiometer (model TZG, Jiangyin No. 2 Medical Equipment Factory, Jiangsu, China), without shoes, to the nearest 0.1 cm. Weight was measured using a uniform and calibrated electronic weight scale (model RGT-140, Shanghai Dachuan Electronic Weighing Apparatus Co. Ltd., Shanghai, China) to the nearest 0.1 kg. When measuring weight, participants were required to wear only light clothing and no shoes. Both height and weight were measured twice, and the mean values were recorded. Smoking was measured by the item “Do you smoke?“ and coded as “0 = never, 1 = currently smoking or have quit smoking”. Alcohol use was measured by the item “Do you drink alcohol?“ and coded as “0 = never, 1 = currently drinking or have quit drinking”.

### Blood pressure

Blood pressure was measured at least twice after 5 min of rest in the sitting position using an auscultatory mercury sphygmomanometer. The average of the two recorded measurements was used. Hypertension was defined as having a systolic blood pressure (SBP) ≥ 140 mm Hg or diastolic blood pressure (DBP) ≥ 90 mm Hg or the use of antihypertensive medication, whereas prehypertension was defined as having an SBP ≥ 130 mm Hg and < 140 mmHg or DBP ≥ 85 mmHg and < 90 mm Hg.

### Physical examination and body composition

We measured adults’ body composition using professional medical personnel with a GE Healthcare Lunar iDXA dual-energy X-ray bone densitometer, in accordance with the standard use process and program requirements described by the instrument, we scanned the whole body and collected images. The participants were placed as required, lying flat on the scanning bed, with the body in the middle of the instrument, with the thumb facing up, and the palm facing, but not touching, the leg. All measurements were carefully checked before the examination. During each on-site physical examination, a special person was assigned to conduct on-site supervision to ensure that the measurement methods and records of each measurement index were correct and standardized.

TSM was computed based on age and appendicular LBM according to the existing equations with high validity in Chinese adults samples [18]. Other regional LBM-related indices were computed as legs, arms, and trunk LBM. Android to gynoid fat ratio (AOI) was calculated as android fat mass divided by gynoid fat mass, which is a main indicator of body fat distribution or central obesity [8, 19, 20].

### Statistical analysis

Descriptive statistics (e.g., means and standard deviations for continuous variables, frequency for categorical variables) were calculated for all variables in this study. Differences by BP categories (i.e., normal, prehypertension, and hypertension) were examined by analysis of variance (ANOVA) tests for continuous variables and Pearson’s chi-squared test for categorical variables.

We used a generalized linear model (GLM) adjusting for age, sex, height, weight, smoking, and alcohol use. The roles of sex and age were also analyzed. Then, the continuous body fat composition were categorized into two groups, using the 50th percentile as a cutoff point (the low-level group vs. high-level group). The cutoff values were 20.00 for BF and 0.53 for AOI. We assessed the effects of each LBM index (independent variable) on blood pressure (dependent variable) separately in the low-level group and the high-level group of body fat composition (stratification variables) after adjustment for confounders. Furthermore, we examined the role of age distribution on these associations.

All analyses were performed using R (version 4.0.3). The “glm” and “psych” were used to fit the GLM model. Statistical significance was defined as a two-sided p-value of less than 0.05. The “ggplot2” and “cowplot” were used to draw the figures.

## Results

### Study population

Table 1 showed the characteristics of the study population. The mean age was 38.91 (SD: 15.94) years for the full population, and mean age of the hypertension group (50.36 ± 14.22) was significant higher than that of the prehypertension group (38.44 ± 15.98) and the normal group (30.05 ± 14.45) (*p* < 0.0001). And males had higher rates of hypertension (11.2% vs. 4.9%) and prehypertension (21.6% vs. 14.6%) than females with a statistically significant difference (*p* < 0.001).

The mean SBP and DBP were 122.04 ± 15.98 mmHg and 76.68 ± 10.59 mmHg. The mean TSM or LBM were higher in participants with hypertension and prehypertension than in those without (*ps* < 0.001). Also, there were significant differences in BMI, height, weight, smoking, alcohol use, and other sociodemographic factors based on BP category (*ps* < 0.001).

**Table 1:** Characteristics of participants

		Total N = 1465	Normal, n = 664	Prehypertension, n = 556	Hypertension, n = 245	*p*
Age, y		38.91(15.94)	35.03(14.45)	38.44(15.98)	50.36(14.22)	< 0.001
Male, n (%)		742(50.6%)	217(14.8%)	361(21.6%)	164(11.2%)	< 0.001
BMI, kg/m^2^		23.60(4.33)	22.18(3.59)	24.01(4.20)	26.55(4.81)	< 0.001
Height, cm		166.36(7.48)	164.49(7.01)	168.39(7.62)	166.86(7.22)	< 0.001
Weight, kg		65.47(13.44)	60.10(10.81)	68.18(13.19)	74.00(14.41)	< 0.001
Smoking, n (%)		296(20.1%)	83(12.5%)	121(21.8%)	92(37.6%)	< 0.001
Alcohol use, n (%)		504(34.3%)	184(27.7%)	203(36.5%)	117(47.8%)	< 0.001
Antihypertension drugs		/	/	/	48(19.6%)	
**Body composition**						
	FM, kg	20.01(8.01)	18.50(6.79)	19.96(8.30)	24.26(8.85)	< 0.001
	AOI	0.52(0.20)	0.44(0.15)	0.55(0.2)	0.68(0.21)	< 0.001
	TSM, kg	22.19(5.84)	19.99(4.90)	23.88(5.94)	24.20(6.10)	< 0.001
	Arm LBM, kg	4.64(1.43)	4.01(1.20)	5.08(1.41)	5.34(1.38)	< 0.001
	Leg LBM, kg	14.65(3.48)	13.4(2.93)	15.65(3.49)	15.77(3.69)	< 0.001
	Trunk LBM, kg	20.60(4.15)	18.89(3.5)	21.77(4.01)	22.56(4.31)	< 0.001
	Body LBM, kg	43.16(9.09)	39.45(7.65)	45.85(8.86)	47.08(9.39)	< 0.001
**Blood pressure, mmHg**						
	Systolic	122.04(15.98)	108.94(7.16)	126.68(5.86)	146.91(13.16)	< 0.001
	Diastolic	76.68(10.59)	69.39(5.69)	78.39(6.69)	92.52(8.91)	< 0.001

Note: ANOVA for continuous variables and χ^2^ test for categorical variables

### Association of LBM and adults blood pressure with different age groups and sex

As shown in Table 2, significant associations were observed between some LBM indicators and the odds of SBP after adjusting for cofounders. There were no significant associations between DBP and LBM indexes, including TSM, arms LBM, legs LBM, and trunk LBM (all *p* > 0.05). Arm LBM (β = 1.86, 95% CI: 0.77, 2.94) and trunk LBM (β = 0.37, 95% CI: 0.01, 0.73) were significantly associated with SBP.

**Fig. 2:**
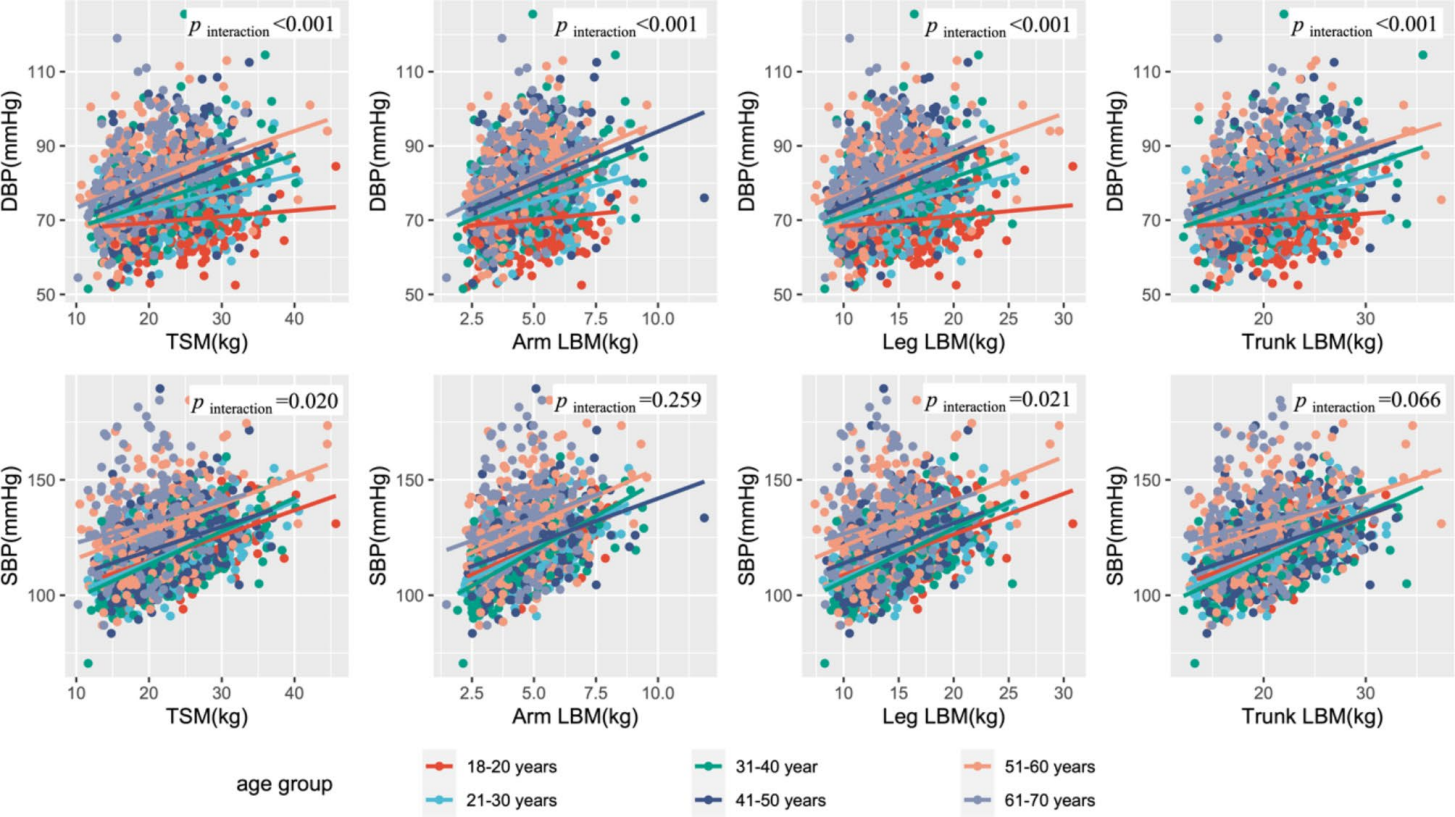
Association of LBM and blood pressure in different age groups

Further tests of the interaction of age and sex were shown in Table S1 and Table S2. The results found that the effect of LBM on DBP was interactive at different ages (*p* < 0.001), and the association of LBM indexes on DBP was stronger in females than males (*p* < 0.001). In order to visualize the role of age, the continuous variable age was transformed into categorical variables. A scatter diagram and its fitted lines showed the visual association between the LBM and blood pressure with different age groups (Fig. 2). With the increase of age group, the influence of LBM indexes including TSM, arms LBM, legs LBM, and trunk LBM on DBP increases gradually, especially after twenty years old. The beta distributions of LBM on SBP and DBP in different age and sex were shown in Table S4.

**Fig. 3:**
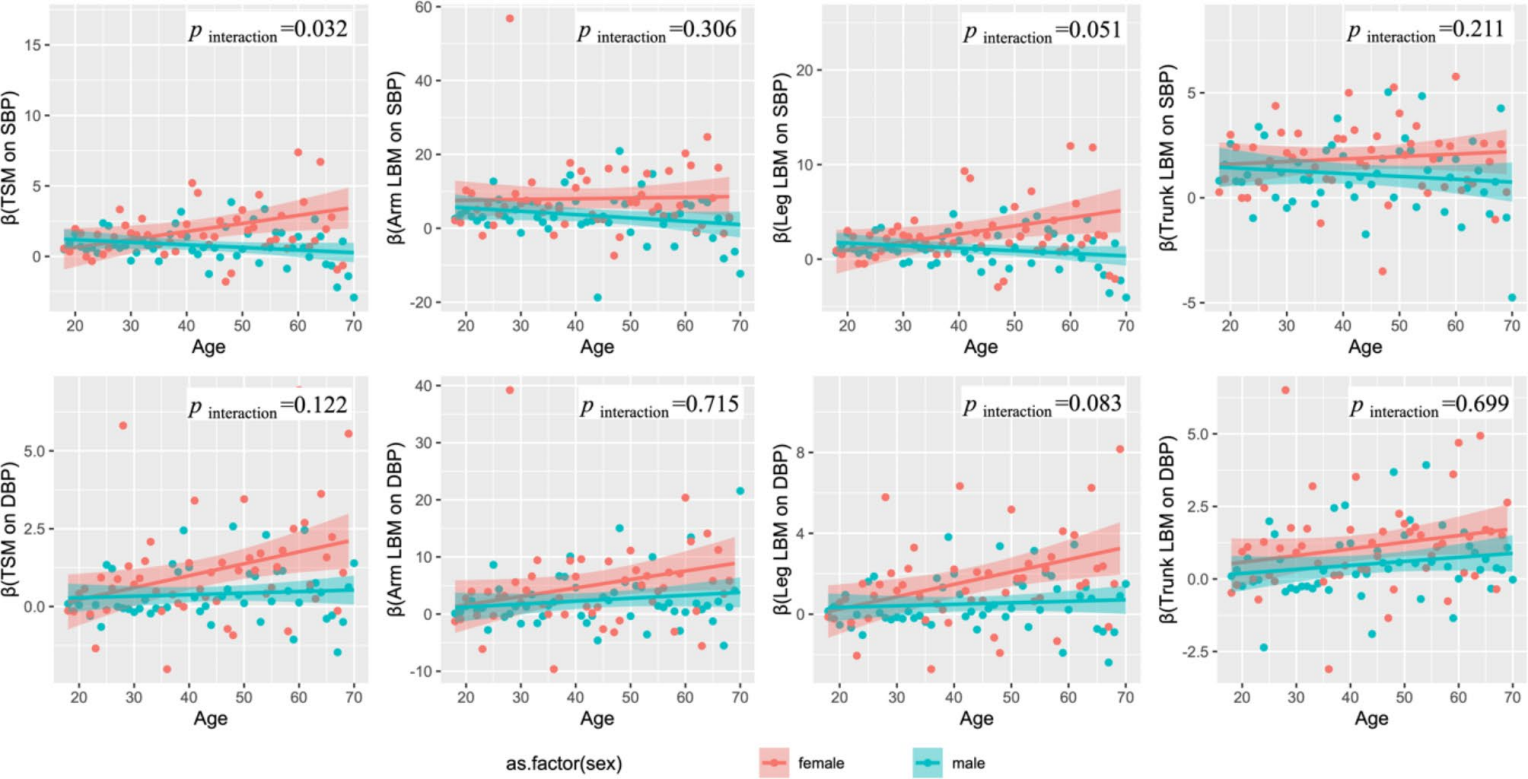
The ages variation tendency of LBM on blood pressure (β) by different gender

We further examined the sex differences in age changes in the relationships described above. As shown in Fig. 3, for females, the influence of LBM indexes on blood pressure increases with age. But for males, the effect of LBM on blood pressure levelled off or declined with age.

### Association of LBM indexes and blood pressure modified by body fat

As shown in Table 2, a difference was witnessed between adults’ LBM and blood pressure when stratified by body fat. To enhance interpretability, a stratification analysis based on the low and high level of FM and AOI was performed (Table S3). Association between adults’ arms LBM and SBP in the high-level FM group was observed (β = 2.74, 95%CI: 0.78, 4.70), while there was no significant association between adults’ arms LBM and SBP in the low-level FM group (β = 1.30, 95% CI: -0.11, 2.71). While comparing the low and high level AOI group, the association between adults’ arms LBM and SBP was smaller in the latter (1.80 [95% CI: 0.18, 3.58] vs. 2.08 [95% CI: 0.74, 3.47]). For legs LBM, it showed the same tendency in SBP. Adults’ legs LBM was significantly associated with SBP in the high-level AOI group (β = 0.65, [95% CI: 0.08, 1.23]), while there was no significant association between adults’ legs LBM and SBP in the low-level AOI group ((β = 0.14, 95% CI: -0.63, 0.91).

**Fig. 4:**
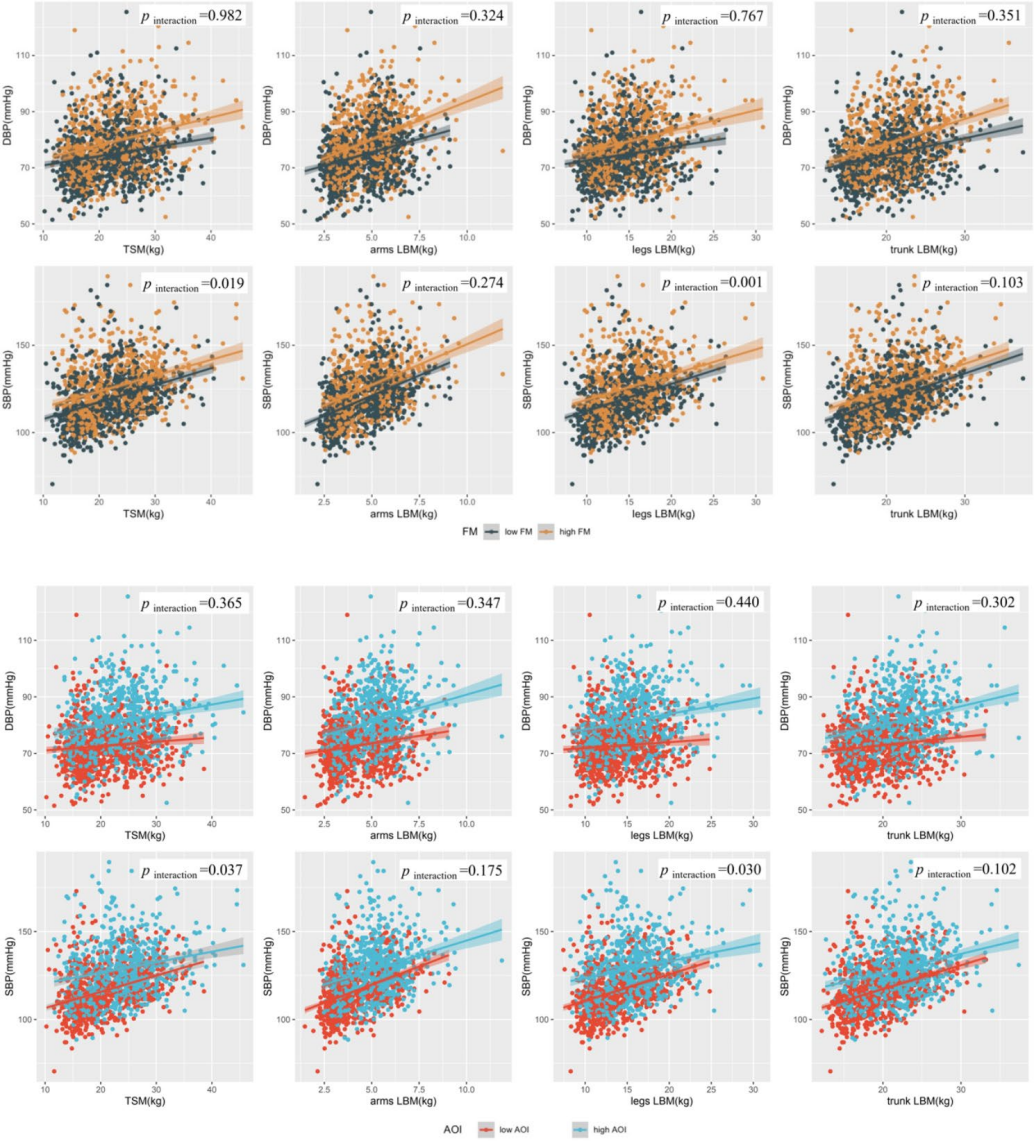
Associations of LBM and blood pressure in different FM and AOI groups

The interactions of TSM with both FM and AOI on SBP were shown in Table S3. The interactions between FM and TSM (*p* = 0.019), between FM and legs LBM (*p* = 0.001) on SBP were significant. Additionally, the interaction between AOI and TSM (*p* = 0.037), between FM and legs LBM (*p* = 0.030) on SBP were also significant. High FM/AOI and LBM were associated with higher SBP. The visual results presentation of the modified effects by FM and AOI were shown in Fig. 4. We further examined the ages variation tendency of LBM indexes on BP (β) in different FM and AOI groups, as depicted in Fig. S1 and Fig. S2.

**Table 2:** Association between LBM and blood pressure (regression coefficient, β, 95% CI) by low- and high-level group of body fat

		Combined		Low FM	High FM		Low AOI	High AOI
DBP	TSM	-0.10(-0.30,0.10)		-0.08(-0.34,0.18)	-0.04(-0.45,0.37)		-0.09(-0.35,0.17)	-0.04(-0.36,0.28)
	Arms LBM	0.18(-0.55,0.92)		-0.16(-1.15,0.83)	1.04(-0.24,2.32)		0.28(-0.71,1.27)	0.36(-0.74,1.46)
	Legs LBM	-0.29(-0.61,0.02)		-0.16(-0.57,0.24)	-0.45(-1.09,0.18)		-0.23(-0.64,0.18)	-0.27(-0.77,0.23)
	Trunk LBM	-0.03(-0.28,0.21)		-0.04(-0.37,0.29)	0.22(-0.28,0.72)		-0.17(-0.51,0.16)	0.16(-0.21,0.52)
SBP	TSM	0.27(-0.03,0.57)		0.18(-0.19,0.56)	0.49(-0.14,1.12)		0.24(-0.25,0.73)	0.44(0.07,0.80)
	Arms LBM	1.83(0.75,2.92)		1.30(-0.11,2.71)	2.74(0.78,4.70)		1.80(0.18,3.58)	2.08(0.70,3.47)
	Legs LBM	0.34(-0.13,0.81)		0.29(-0.29,0.86)	0.46(-0.51,1.43)		0.14(-0.63,0.91)	0.65(0.08,1.23)
	Trunk LBM	0.38(0.01,0.74)		0.46(-0.01,0.93)	0.34(-0.43,1.11)		0.34(-0.23,0.90)	0.46(-0.02,0.93)

Note: All the models were controlling for age, sex, height, weight, smoking and alcohol use

## Discussion

Previous studies focusing on the relationship between body composition and blood pressure have overemphasized the harmful effects of body fat and obesity, while ignoring the effect of LBM as a higher percentage of body weight. Our study found that TSM and local LBM indexes, especially arms LBM, were significantly associated with SBP and showed an interaction with fat distribution, with some differences in age groups and sex.

A low proportion of LBM is generally considered to be a risk factor for morbidity and mortality in old age [13]. However, recent studies have shown opposite results, showing a positive association between LBM and several cardiovascular abnormalities, including hypertension [21, 22]. Our results also demonstrated significant associations between LBM in the arms and trunk, and elevated SBP in Chinese adults. Additionally, the associations of LBM on the odds of blood pressure increases with age, especially in females. Although several studies have found that male athletes have higher blood pressure than female athletes [23]. Given that LBM has a greater effect on the growth of blood pressure in adult women, we still need to be concerned about health issues associated with increased LBM in older women.

This study also found interactions between fat composition and LBM that influences blood pressure. For adults with high FM or AOI, arms LBM has a stronger positive association SBP. Previous researches on the associations between obesity and blood pressure have been based on BMI [24, 25], which does not distinguish between fat mass and LBM. This study suggests that both body fat and LBM including TSM should be considered in adults blood pressure prevention and control.

To our knowledge, the mechanism by which LBM is positively correlated with blood pressure is not clear. One possible explanation is the role of carotid intima-media thickness (c-IMT). Studies have found that the increase of LBM is significantly correlated with the rise of c-IMT [21]. Multiple epidemiological studies have shown that obesity and central obesity are associated with c-IMT [26, 27]. Hypertension is the most common risk factor for cardiovascular disease among elite athletes. Physical services, such as exercise training, require increased cardiac output and may promote the development of left ventricular hypertrophy (LVH), which has been linked to elevated blood pressure [28]. Another possible explanation is that physical activity associated with skeletal muscle hypertrophy (e.g. resistance training) can increase arterial stiffness and activate the sympathetic nervous system [29], which may increase blood pressure in humans [30]. In a previous report on athletes, it was found that blood pressure values were higher in strength training athletes than in endurance training athletes, which could be attributed to larger LBM [28]. In addition, an increase in LBM represents the increase of muscle fibers. Type II fibers, which make up a larger proportion of muscle fibers in muscle hypertrophy, have been shown to be positively correlated with resting blood pressure [31]. On the other hand, skeletal muscle cells have also been found to produce and secrete cytokines that are associated with inflammation, which can contribute to the development of inflammation-related diseases [32]. Therefore, our results support these conjectures and explorations.

In the present study, our results show that TSM and arm LBM are positively correlated with increased blood pressure in Chinese adults, based on the measurement of DXA. And these associations are more obvious in females and in group with high FM and high AOI groups as age increases. These results remind us that we should be aware of the potential negative effects of skeletal muscle elevation or muscle gain. Although physical exercise is an essential component of a healthy lifestyle, it is important to note that exercise, particularly in the arms, can have a significant impact on blood pressure, especially SBP. Skeletal muscle and fat status in the elderly, particularly in females, should be the focus of intervention for blood pressure control.

Body composition is a better indicator of fat and non-fat distribution than BMI. The study had significant advantages, including its advanced assessment of body composition (DXA) [16], large sample size, and a wide age range of Chinese adults. Some potential limitations should also be noted in this study. Firstly, the study has a cross-sectional design, which means it is unable to explore the causality between LBM indexes and BP. Future studies can be improved by utilizing queue data. Secondly, although peripheral blood pressure (PBP), which is measured using a mercury sphygmomanometer to monitor vascular pulsation at peripheral sites, has long been widely accepted, new evidence suggests that central artery and venous BP waveforms are significantly more associated with cardiovascular events than PBP [33, 34]. It is not entirely clear whether the brachial artery blood pressure measured indirectly reflects the true central artery blood pressure in individuals with varying skeletal muscle mass. Future studies may focus on more accurate, non-invasive central blood pressure waveform monitoring [35, 36]. Thirdly, the sample of this study is from Beijing, which cannot completely represent the overall situation of Chinese adult population. Future studies could sample across the country to give a more comprehensive picture of the current state of health problems in China and further explore regional health equity issues.

## Conclusion

Although LBM and increased muscle strength are considered key factor in preventing age-related problems, the potential negative impact of increased LBM on blood pressure cannot be ignored. This study found that an increase in skeletal muscle mass, especially in the upper extremities, may have adverse effects on blood pressure. The association was found to be more pronounced in females and individuals with high FM and high AOI as they aged. The gold standard for DXA is used to distinguish the different effects of LBM on blood pressure in different fat distribution groups, which is of great significance for the profound understanding of the association between obesity and hypertension. In addition to controlling fat content, future LBM gain from should be considered as part of a policy to strengthen adult cardiovascular health systems, which includes not overly focusing on physical exercise, particularly upper body training.

### Electronic supplementary material

Below is the link to the electronic supplementary material.

Supplementary Material 1

## Data Availability

The datasets used and/or analyzed during the current study are available from the corresponding author on reasonable request.
