# Supplementary material for: Lean body mass positively associate with blood pressure in Chinese adults: the roles of ages and body fat distribution
Source: BMC Public Health. 2023 Dec 7;23:2453. doi: 10.1186/s12889-023-17312-0 (PMC10704775; doi:10.1186/s12889-023-17312-0)
Supplement: Supplementary file 1 — Supplementary Material 1 [file 12889_2023_17312_MOESM1_ESM.pdf]

Supporting Information to:

Lean Body Mass Positively Associate with Blood Pressure in Chinese Adults: The Roles of Ages and Body Fat Distribution

List of Tables and Figures:

Table S1 Association and interaction between age and LBM on adults' blood pressure (regression coefficient, β)

Table S2 Association and interaction between sex and LBM on adults' blood pressure (regression coefficient, β)

Table S3 Association and interaction between body fat and LBM on adults' blood pressure (regression coefficient, β)

Fig. S1 The ages variation tendency of LBM indexes on BP (β) in different FM groups

Fig. S2 The ages variation tendency of LBM indexes on BP (β) in different AOI groups

Table S4 The beta distributions of LBM on SBP and DBP in different age and sex

Table S1 Association and interaction between age and LBM on adults' blood pressure (regression coefficient, β)

Factors		SBP			DBP		
		Est.	Std.	*p* for interaction	Est.	Std.	*p* for interaction
Model of age & TSM	Age	0.02	0.00	**0.020**	0.02	0.00	**<0.001**
	TSM	0.22	0.08		-0.30	0.08	
Model of age & Arms LBM	Age	0.02	0.00	0.259	0.02	0.00	**<0.001**
	TSM	0.22	0.07		-0.20	0.07	
Model of age & Legs LBM	Age	0.02	0.00	**0.021**	0.02	0.00	**<0.001**
	Legs LBM	0.19	0.07		-0.33	0.07	
Model of age & Trunk LBM	age	0.02	0.00	0.066	0.02	0.00	**<0.001**
	Trunk LBM	0.21	0.08		-0.26	0.08	

Note: The regression coefficient of β was calculated using the general linear model. All the models were controlling for sex, height, weight, smoking, alcohol use.

Table S2 Association and interaction between sex and LBM on adults' blood pressure (regression coefficient, β)

Factors		SBP			DBP		
		Est.	Std.	*p* for interaction	Est.	Std.	*p* for interaction
Model of sex & TSM	Sex	-0.44	0.09	0.602	-0.38	0.09	**0.018**
	TSM	0.05	0.10		-0.26	0.10	
Model of sex & Arms LBM	Sex	-0.29	0.09	**0.029**	-0.31	0.10	0.111
	TSM	-0.04	0.11		-0.13	0.11	
Model of sex & Legs LBM	Sex	-0.47	0.08	0.692	-0.31	0.10	**0.023**
	Legs LBM	0.04	0.09		-0.13	0.11	
Model of sex & Trunk LBM	sex	-0.45	0.08	0.477	-0.38	0.08	0.365
	Trunk LBM	0.04	0.10		-0.09	0.10	

Note: The regression coefficient of β was calculated using the general linear model. All the models were controlling for age, height, weight, smoking, alcohol use.

Table S3 Association and interaction between body fat and LBM on adults' blood pressure(regression coefficient, β)

Factors		SBP Est.	SBP Std.	SBP *p* for interaction	DBP Est.	DBP Std.	DBP *p* for interaction
Model of FM & TSM	FM	0.26	0.29	**0.019**	-0.12	0.20	0.982
	TSM	0.38	0.35		-0.25	0.24	
Model of FM & Arms LBM	FM	0.26	0.22	0.274	0.01	0.15	0.324
	Arms LBM	2.77	0.99		0.23	0.67	
Model of FM & Legs LBM	FM	0.28	0.28	**0.001**	-0.17	0.19	0.767
	Legs LBM	0.50	0.48		-0.64	0.32	
Model of FM & Trunk LBM	FM	0.28	0.33	0.103	-0.06	0.22	0.351
	Trunk LBM	0.53	0.44		-0.01	0.30	
Model of AOI & TSM	AOI	20.00	7.71	**0.037**	3.84	5.17	0.365
	TSM	0.66	0.23		-0.16	0.16	
Model of AOI & Arms LBM	AOI	13.54	6.96	0.175	4.34	4.67	0.347
	Arms LBM	2.81	0.88		-0.16	0.59	
Model of AOI & Legs LBM	AOI	21.79	8.25	**0.030**	4.03	5.52	0.440
	Legs LBM	1.02	0.38		-0.36	0.25	
Model of AOI & Trunk LBM	AOI	19.32	9.40	0.102	2.09	6.30	0.302
	Trunk LBM	0.78	0.30		-0.17	0.20	
Model of FM & TSM	FM	0.26	0.29	**0.019**	-0.12	0.20	0.982
	TSM	0.38	0.35		-0.25	0.24	
Model of FM & Arms LBM	FM	0.26	0.22	0.274	0.01	0.15	0.324
	Arms LBM	2.77	0.99		0.23	0.67	
Model of FM & Legs LBM	FM	0.28	0.28	**0.001**	-0.17	0.19	0.767
	Legs LBM	0.50	0.48		-0.64	0.32	
Model of FM & Trunk LBM	FM	0.28	0.33	0.103	-0.06	0.22	0.351
	Trunk LBM	0.53	0.44		-0.01	0.30	

Note: All the models were controlling for age, gender, height, weight, smoking, alcohol use.

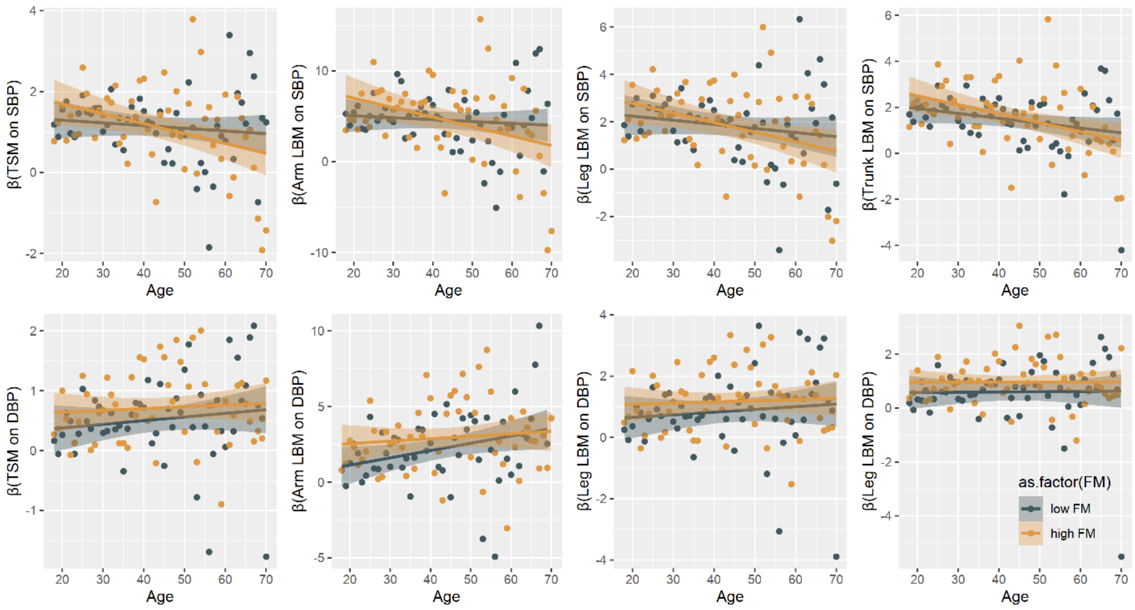

Fig. S1 The ages variation tendency of LBM indexes on BP(β) in different FM groups

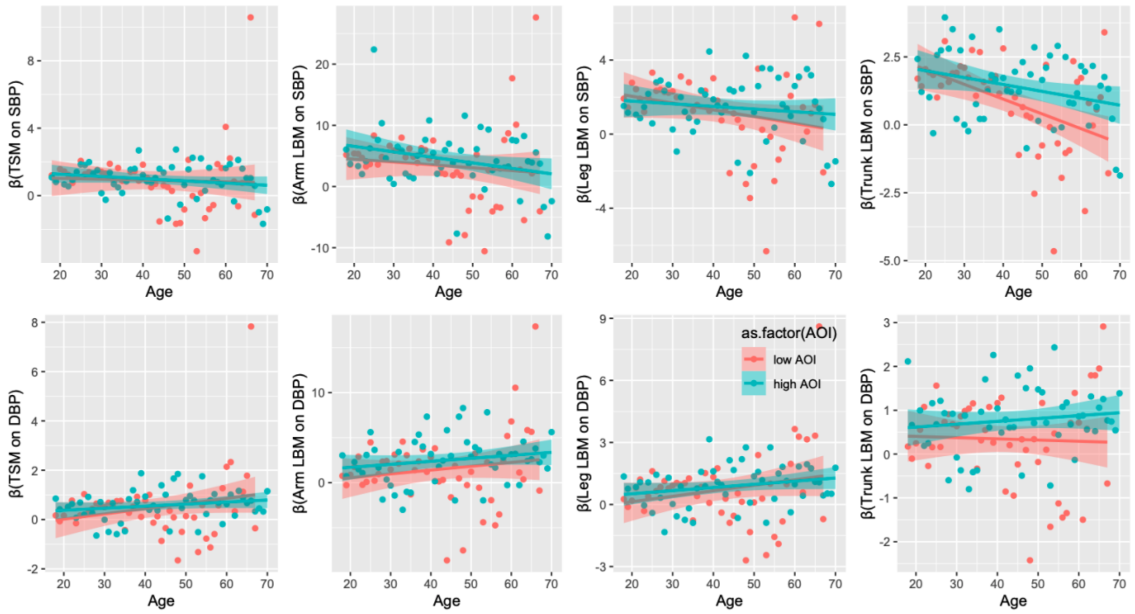

Fig. S2 The ages variation tendency of LBM indexes on BP (β) by different AOI groups

Table S4 The beta distribution of LBM on SBP and DBP in different age and gender

Sex	Age	SBP								DBP							
		TSM		LBM_arms		LBM_legs		LBM_trunk		TSM		LBM_arms		LBM_legs		LBM_trunk	
Male	18	110.72	0.50	110.02	2.62	111.67	0.71	105.84	0.81	68.53	0.09	70.26	0.12	68.15	0.15	68.73	0.09
	19	98.85	0.94	103.21	3.75	98.38	1.47	87.93	1.58	64.55	0.21	67.88	0.44	63.67	0.38	63.15	0.31
	20	78.06	1.60	92.45	4.77	73.78	2.77	60.29	2.57	71.16	0.05	64.60	1.37	81.20	-0.53	78.13	-0.25
	21	108.05	0.68	108.25	3.19	107.62	1.09	108.09	0.79	59.77	0.57	62.03	2.32	58.60	0.96	58.70	0.71
	22	112.04	0.46	112.70	2.01	113.01	0.66	106.99	0.75	81.95	-0.30	70.16	0.70	85.29	-0.66	76.22	-0.09
	23	104.98	0.62	104.61	3.05	105.94	0.90	96.59	1.09	73.15	0.05	68.57	1.07	75.02	-0.02	75.38	-0.03
	24	115.06	0.24	116.45	0.90	114.65	0.40	143.98	-0.97	90.48	-0.65	88.88	-2.79	90.75	-1.03	127.62	-2.36
	25	65.34	2.36	56.23	12.70	75.66	3.06	49.90	3.38	45.08	1.34	31.94	8.63	53.00	1.61	34.73	1.99
	26	65.21	2.16	76.73	7.80	66.67	3.32	54.18	2.97	42.13	1.22	48.82	4.34	42.87	1.87	38.82	1.54
	27	102.62	0.81	106.26	3.27	102.33	1.27	88.30	1.57	75.67	0.00	78.33	-0.50	74.33	0.07	59.84	0.68
	28	105.71	0.60	110.36	2.05	103.72	1.04	121.90	0.01	76.35	-0.06	74.54	0.05	77.89	-0.17	85.09	-0.44
	29	101.88	0.76	93.29	5.04	108.48	0.79	93.75	1.20	79.97	-0.08	70.54	1.32	82.14	-0.26	84.92	-0.31
	30	135.76	-0.31	134.64	-1.25	135.56	-0.46	139.07	-0.49	82.92	-0.18	87.94	-1.67	80.58	-0.15	86.86	-0.37
	31	113.16	0.27	79.93	7.49	125.12	-0.29	124.54	-0.18	76.27	-0.07	67.63	1.27	78.25	-0.22	80.59	-0.26
	32	91.98	1.11	80.15	7.23	96.15	1.48	83.86	1.68	70.90	0.22	57.42	3.41	74.36	0.14	82.31	-0.25
	33	102.76	0.78	106.35	2.98	102.67	1.22	103.53	0.88	87.71	-0.22	91.12	-1.60	84.93	-0.19	89.29	-0.33
	34	110.00	0.53	114.65	1.66	107.58	0.98	104.64	0.83	78.76	-0.06	79.47	-0.37	78.38	-0.07	80.97	-0.15
	35	134.93	-0.35	126.35	-0.13	136.89	-0.65	132.41	-0.29	82.13	-0.11	70.81	1.50	85.80	-0.38	72.48	0.29
	36	117.23	0.12	101.61	2.96	127.94	-0.39	114.59	0.25	78.05	-0.02	63.66	2.13	87.07	-0.52	86.67	-0.38
	37	86.75	1.57	91.84	6.15	88.49	2.37	74.53	2.26	44.05	1.36	40.59	6.66	50.54	1.78	21.74	2.45
	38	58.90	2.34	50.33	12.45	71.53	2.92	59.98	2.51	50.75	1.11	43.21	6.53	57.96	1.32	52.05	1.16
	39	44.11	3.17	44.89	14.41	47.81	4.77	36.74	3.78	15.76	2.45	22.92	10.10	16.18	3.82	20.10	2.54

40	85.78	1.40	81.12	7.36	89.07	1.98	77.25	1.99	45.79	1.26	52.86	4.62	45.26	2.00	50.86	1.23
41	115.03	0.43	120.01	1.04	113.93	0.75	111.45	0.62	76.52	0.30	86.77	-0.29	67.96	0.98	80.93	0.16
42	116.24	0.15	110.02	1.71	118.74	0.08	119.40	0.03	83.81	-0.15	88.94	-1.56	81.64	-0.11	93.82	-0.59
43	104.20	0.63	108.36	2.01	101.91	1.16	97.08	1.00	78.27	0.01	80.58	-0.31	76.43	0.13	74.44	0.18
44	160.05	-1.25	231.16	-18.73	150.50	-1.36	167.20	-1.74	100.55	-0.59	110.79	-4.61	97.89	-0.76	127.74	-1.90
45	114.90	0.60	112.14	3.24	117.92	0.75	115.62	0.63	78.91	0.21	67.30	3.00	84.91	-0.04	61.77	0.96
46	123.65	-0.06	113.43	1.53	127.08	-0.30	94.57	1.15	74.10	0.33	69.07	2.40	77.98	0.28	69.28	0.56
47	61.37	2.09	73.08	7.49	74.03	2.51	70.83	1.86	62.43	0.56	55.21	3.79	69.30	0.46	68.61	0.35
48	26.18	3.85	1.37	20.92	40.47	5.23	7.35	5.03	18.23	2.57	-4.66	15.03	30.00	3.37	-1.97	3.68
49	129.45	0.05	93.24	6.55	146.60	-0.99	130.34	0.02	78.67	0.37	55.96	5.59	89.97	-0.13	74.75	0.57
50	111.75	0.52	132.42	-1.08	104.09	1.26	73.39	2.22	80.90	0.11	86.06	-0.36	78.88	0.29	48.29	1.51
51	89.11	1.61	81.14	7.80	91.75	2.43	60.30	2.84	57.08	1.05	52.85	4.94	58.53	1.60	33.83	2.03
52	60.15	2.64	58.85	11.93	63.30	3.96	78.06	2.24	57.90	0.97	55.34	4.73	60.21	1.40	71.85	0.54
53	141.10	-0.47	158.58	-4.99	135.96	-0.42	139.49	-0.44	94.39	-0.49	103.04	-3.57	92.11	-0.63	97.99	-0.69
54	55.24	3.36	57.86	14.70	64.99	4.56	24.78	4.85	35.71	2.30	38.03	9.99	42.28	3.14	0.68	3.93
55	121.19	0.62	107.51	5.23	123.85	0.80	106.45	1.32	84.44	0.17	80.61	1.44	85.19	0.21	74.89	0.59
56	89.06	1.72	126.38	1.10	80.75	3.15	88.14	1.87	51.57	1.13	73.65	1.18	46.79	2.04	53.82	1.12
57	82.11	1.67	105.67	3.18	80.28	2.71	79.08	1.89	50.52	1.15	67.95	1.96	48.69	1.89	35.17	1.85
58	156.82	-0.87	162.60	-4.94	152.22	-1.07	149.75	-0.68	72.01	0.50	83.07	0.48	63.40	1.25	75.36	0.42
59	126.99	0.60	119.28	3.99	129.26	0.78	119.16	0.97	111.81	-1.06	102.05	-2.93	116.35	-1.91	117.00	-1.35
60	96.21	1.40	94.60	6.64	94.72	2.22	88.01	1.83	81.96	0.13	83.49	0.34	81.40	0.22	85.39	0.01
61	124.80	0.62	105.51	6.18	128.25	0.73	170.60	-1.41	29.47	2.46	12.62	13.45	34.64	3.45	49.90	1.61
62	122.07	-0.04	127.82	-1.24	119.32	0.13	110.91	0.46	65.39	0.54	68.09	1.87	64.25	0.90	52.66	1.13
63	72.37	2.63	93.26	7.75	69.13	4.23	71.91	2.69	70.45	0.76	84.47	0.80	67.50	1.35	67.15	0.91
64	97.67	1.42	92.15	7.03	101.12	1.95	101.16	1.30	70.38	0.46	72.96	1.48	69.13	0.78	73.82	0.32

	65	151.01	-0.56	152.90	-2.65	150.96	-0.86	131.41	0.26	98.59	-0.39	96.45	-1.26	100.46	-0.72	73.08	0.68
	66	157.83	-0.65	128.63	2.64	168.01	-1.68	160.41	-0.77	91.12	-0.28	72.77	2.20	97.52	-0.86	92.21	-0.33
	67	193.97	-2.20	184.60	-8.20	198.12	-3.58	114.20	0.76	124.57	-1.47	118.59	-5.52	127.25	-2.39	74.61	0.38
	68	106.18	1.06	116.06	2.68	105.05	1.68	34.60	4.26	93.07	-0.49	75.75	1.22	95.23	-0.89	75.05	0.31
	69	168.70	-1.40	170.08	-6.32	170.88	-2.25	157.17	-0.94	63.33	0.62	55.50	4.10	63.91	0.91	52.57	1.08
	70	209.73	-2.91	207.10	-12.29	205.10	-4.05	245.71	-4.75	55.47	1.39	-25.17	21.56	64.39	1.50	87.93	-0.02
	18	100.58	0.56	103.97	2.12	99.51	0.89	106.13	0.27	70.22	-0.13	71.86	-1.27	69.47	-0.13	76.00	-0.48
	19	106.56	0.34	107.73	1.53	106.22	0.51	97.24	0.90	72.49	-0.16	72.50	-0.89	72.44	-0.23	73.20	-0.21
	20	73.69	1.96	74.58	10.25	71.96	3.00	58.65	3.00	62.08	0.42	60.28	2.76	62.52	0.57	53.80	0.94
	21	86.51	1.64	84.02	9.48	86.57	2.40	73.31	2.41	60.55	0.56	57.38	3.92	61.32	0.76	50.97	1.10
	22	109.62	-0.02	93.81	5.05	114.91	-0.47	109.36	-0.01	73.04	-0.25	69.48	-0.24	73.70	-0.41	68.72	0.00
	23	116.29	-0.34	116.48	-1.98	116.26	-0.49	110.47	0.00	96.10	-1.34	91.70	-6.12	97.53	-2.05	85.06	-0.71
	24	83.20	1.56	89.95	6.57	80.09	2.51	70.02	2.40	54.84	0.93	59.06	3.87	52.90	1.51	49.87	1.27
	25	103.57	0.14	103.42	0.80	103.61	0.20	93.62	0.75	70.95	-0.11	70.04	-0.30	71.39	-0.19	67.04	0.13
	26	101.69	0.59	99.41	3.74	102.02	0.84	104.23	0.47	63.97	0.58	64.53	2.92	63.22	0.92	76.40	-0.08
Female	27	84.73	1.38	88.91	5.89	82.88	2.20	79.03	1.76	56.25	0.88	61.04	3.20	54.24	1.47	53.84	1.06
	28	46.92	3.33	-60.97	56.81	69.64	2.88	26.94	4.38	-35.41	5.81	-48.32	39.20	-5.11	5.79	-49.96	6.51
	29	70.64	2.20	79.74	9.34	67.68	3.47	55.78	3.11	50.13	1.30	55.11	5.59	48.56	2.03	42.74	1.76
	30	80.48	1.64	86.61	6.70	78.98	2.53	72.81	2.14	59.40	0.71	64.73	2.04	57.87	1.17	56.28	0.91
	31	75.33	1.52	78.14	7.51	73.57	2.35	70.02	1.92	52.29	0.91	55.29	4.16	50.67	1.45	49.32	1.14
	32	57.33	2.68	63.67	12.44	54.90	4.09	50.39	3.06	43.68	1.46	47.73	6.61	42.14	2.25	38.89	1.73
	33	82.30	1.52	85.04	7.03	80.62	2.39	71.95	2.18	36.66	2.08	41.06	9.43	34.01	3.29	18.80	3.19
	34	92.41	1.07	96.50	4.30	89.68	1.81	91.07	1.17	63.87	0.49	66.76	1.70	62.01	0.87	63.17	0.54
	35	101.85	0.84	96.01	6.10	102.57	1.18	91.49	1.46	84.68	-0.15	82.08	-0.04	85.55	-0.29	83.62	-0.10
	36	107.96	0.14	117.04	-1.89	104.38	0.50	132.36	-1.21	109.52	-2.01	106.01	-9.64	106.67	-2.72	128.83	-3.11

37	94.31	1.00	94.56	5.13	93.56	1.55	96.17	0.90	66.26	0.38	63.17	2.81	67.02	0.51	71.16	0.12
38	104.16	0.35	106.40	1.12	103.24	0.59	95.60	0.84	71.91	0.10	73.94	-0.07	71.05	0.23	71.38	0.13
39	89.23	1.55	52.70	17.71	96.89	1.71	66.79	2.82	77.31	-0.01	42.27	9.35	82.56	-0.42	68.68	0.46
40	67.26	2.28	70.42	10.97	64.71	3.58	59.04	2.80	46.98	1.40	49.58	6.57	45.15	2.22	42.37	1.70
41	22.46	5.21	58.19	15.47	4.72	9.31	29.25	5.00	15.64	3.40	40.80	9.61	1.12	6.34	15.86	3.52
42	33.67	4.51	68.50	12.98	11.49	8.55	57.24	3.22	63.67	0.56	69.25	1.29	59.75	1.16	67.63	0.35
43	89.74	1.45	101.22	3.73	82.86	2.78	85.66	1.68	70.45	0.31	74.92	0.19	67.72	0.70	70.16	0.32
44	96.20	0.84	102.88	2.43	94.20	1.42	84.72	1.51	52.86	1.10	68.60	1.24	47.33	2.08	50.67	1.25
45	93.95	1.38	110.96	2.43	87.80	2.53	76.47	2.29	73.39	0.17	86.29	-2.63	67.99	0.68	61.88	0.78
46	75.24	2.50	62.24	16.17	76.56	3.60	68.23	2.93	51.45	1.42	43.87	9.22	52.25	2.04	47.98	1.63
47	140.71	-1.79	135.48	-7.39	144.21	-2.95	170.27	-3.50	82.01	-0.72	80.64	-3.18	83.17	-1.16	92.92	-1.35
48	138.37	-1.20	125.07	-2.41	145.44	-2.35	122.67	-0.36	93.24	-0.92	80.46	-1.14	100.08	-1.91	82.86	-0.36
49	78.04	2.64	66.55	15.93	106.71	1.70	29.25	5.26	57.78	1.16	56.81	5.96	66.34	1.06	37.53	2.25
50	62.77	3.29	93.74	7.12	55.55	5.54	48.47	4.02	15.91	3.45	34.70	11.12	15.79	5.18	42.40	1.90
51	82.15	1.86	89.33	7.01	86.43	2.37	79.20	2.04	46.22	1.56	45.82	7.67	52.40	1.76	45.14	1.62
52	80.88	2.06	84.61	9.07	79.40	3.15	71.84	2.58	56.09	1.16	58.40	5.08	55.16	1.79	44.94	1.79
53	61.73	4.37	80.41	14.81	54.04	7.17	76.70	3.42	58.93	1.71	63.14	6.67	59.23	2.50	62.01	1.51
54	114.24	0.67	109.65	4.40	114.88	0.93	115.87	0.56	72.08	0.42	71.22	2.20	71.75	0.64	72.56	0.38
55	110.89	1.06	107.75	5.86	110.97	1.55	124.24	0.23	67.99	0.61	64.00	3.99	69.00	0.80	64.13	0.81
56	105.65	1.21	114.30	3.40	98.92	2.33	93.89	1.84	60.47	1.27	65.88	4.57	55.79	2.25	63.03	1.10
57	80.69	2.89	74.33	15.53	81.77	4.10	84.82	2.59	49.35	1.80	53.61	7.30	46.90	2.83	58.47	1.22
58	111.34	0.56	108.71	3.40	111.65	0.79	111.85	0.52	89.01	-0.79	86.54	-3.07	91.09	-1.33	88.86	-0.77
59	114.50	1.20	111.29	6.17	119.91	1.30	90.58	2.46	39.39	2.50	54.82	7.07	34.69	4.10	16.88	3.61
60	-10.78	7.38	41.76	20.27	-25.95	11.97	19.33	5.77	-46.57	6.95	-1.67	20.37	-58.48	11.08	-5.76	4.69
61	70.82	3.86	74.92	17.02	66.88	5.88	126.97	0.36	40.55	2.69	40.51	12.72	39.88	3.92	80.37	0.21

62	109.73	0.69	124.55	-0.89	103.64	1.52	106.62	0.86	66.37	0.70	76.31	0.53	61.98	1.40	53.22	1.46
63	104.53	1.10	118.87	0.85	97.30	2.24	110.57	0.68	71.79	0.25	94.55	-5.58	58.76	1.55	73.90	0.11
64	17.30	6.71	40.43	24.72	-8.05	11.80	-12.71	8.54	15.58	3.62	25.20	14.10	3.38	6.25	-6.18	4.94
65	92.45	1.93	95.43	8.22	94.49	2.59	81.02	2.59	51.60	1.58	57.29	5.82	51.58	2.26	49.33	1.70
66	83.50	2.78	71.62	16.40	99.64	2.48	98.20	1.71	41.30	2.23	38.25	11.24	49.98	2.37	48.75	1.63
67	138.84	-0.94	129.25	-1.39	143.22	-1.74	141.86	-1.04	77.78	-0.14	63.40	3.56	82.42	-0.62	81.63	-0.35
68	152.53	-0.65	131.03	3.00	166.06	-2.08	97.66	2.56	61.51	1.09	58.40	5.84	62.11	1.50	53.42	1.51
69	-69.92	16.88	NA	NA	-83.61	25.45	137.82	0.27	6.50	5.55	-40.84	37.59	3.83	8.16	36.96	2.63
70	NA	NA	NA	NA	NA	NA	NA	NA	NA	NA	NA	NA	NA	NA	NA	NA
